# Ultrasonic‐pretreated lipase‐catalyzed synthesis of medium–long–medium lipids using different fatty acids as *sn*‐2 acyl‐site donors

**DOI:** 10.1002/fsn3.1083

**Published:** 2019-06-20

**Authors:** Qiang Wang, Yuejie Xie, David R. Johnson, Yuanyuan Li, Zhifei He, Hongjun Li

**Affiliations:** ^1^ College of Food Science Southwest University Beibei, Chongqing China; ^2^ College of Biological and Chemical Engineering Chongqing University of Education Chongqing China; ^3^ Department of Food Science University of Massachusetts Amherst Massachusetts

**Keywords:** fatty acid, lipase, lipid synthesis, medium–long–medium, ultrasound pretreatment

## Abstract

The current work aimed to evaluate the effect of ultrasonic treatment on the enzymatic transesterification of medium–long–medium (MLM) lipids using 2‐monoacylglycerol, bearing distinct fatty acids at the *sn*‐2 position with palmitic acid, octadecanoic acid, oleic acid, eicosapentaenoic acid, and docosahexaenoic acids as *sn*‐2 acyl donors. The effects of ultrasonic treatment conditions, including substrate concentration, reaction temperature and time, and enzyme loading, on the insertion of fatty acids into the *sn*‐2 acyl position of MLM lipids were investigated. The data showed that low‐frequency ultrasonic treatment could remarkably improve the insertion rate of polyunsaturated fatty acid (PUFA) into the *sn*‐2 position of MLM lipids, compared with the conventional treatment method. By increasing the ultrasonic frequency from 20 to 30 KHz, while maintaining power at 150 W, the rate of synthesis of monounsaturated fatty acid and PUFA increased from 23.7% and 26.8% to 26.6% and 32.4% (*p* < 0.05), respectively. Moreover, ultrasonic treatment reduced the optimum reaction temperature from 45 to 35°C. However, the activity of Lipozyme RM‐IM treated with ultrasound considerably declined from 31.10% to 26.90% (*p* < 0.05) after its fourth cycle, which was lower than that without ultrasonic treatment. This work provokes new routes for the utilization of ultrasonic technology in the synthesis of MLM lipids using different fatty acids as *sn*‐2 acyl donors.

## 1 INTRODUCTION

Structured lipids have specific physicochemical properties or nutritional functions as they contain nutritional functional fatty acids inserted into the desired position of the glycerol backbone by chemical or enzymatic modification of natural oils (Wang et al., 2015; Wang, Xia, Xu, Xie, & Duan, 2012). Construction of structured lipids can be optimized via the incorporation of required or special functional fatty acids into specific locations on the glycerol backbone. Reconstructed lipids produce new physiological functions and nutritional values and play indispensable roles in anabolism and catabolism inside the body (Lee & Akoh, 1998; Michalski et al., 2013). Numerous clinical and nutritional reports have shown that structured lipids can effectively prevent certain diseases by enhancing the absorption and utilization of fatty acids, as well as other types of lipids, reducing serum and cholesterol levels, improving immunity, and preventing obesity, cancer, and nutritional disorders (Michalski et al., 2013; Morales‐Medina et al., 2015; Mu & Høy, 2001).

The synthesis of special structured lipids involves linking fatty acids, including short‐, medium‐, and long‐chain fatty acids, to a glycerol carbon backbone, followed by determination of their preestablished structural composition. Among these structured lipids, medium–long–medium (MLM) lipids have been extensively studied (Akanbi, Adcock, & Barrow, 2013; Nagao et al., 2011; Wang et al., 2015), typically consisting of medium‐chain fatty acids (˂12) located at *sn*‐1 and *sn*‐3 positions, as well as the long‐chain fatty acids at *sn*‐2 position of the glycerol backbone. The lipid structure enables triacylglycerols without a carnitine carrier to directly enter the mitochondrial oxidative metabolism by preventing the long‐chain fatty acids entering the metabolic pathways as controlled by regulator protein and carbohydrate intake, thereby promptly providing energy for the body (Nicholson, Khademi, & Moghadasian, 2013). Additionally, owing to their special structures, MLM lipids are easily digested, quickly absorbed, and utilized (Feltes, Oliveira, Block, & Ninow, 2013). These lipids can enhance the absorption of *sn*‐2 fatty acids into the body, thereby providing timely energy, and reduce blood lipids, as well as benefiting weight loss in certain populations (Feltes et al., 2010; Nagao et al., 2011). Due to these advantages, structured lipids and their preparation have attracted increasing attention from researchers (Caballero, Soto, Olivares, & Altamirano, 2014; Khodadadi, Aziz, St‐Louis, & Kermasha, 2013).

Medium–long–medium lipids are synthesized by various methods. Compared with chemical methods, an enzymatic approach is more widely used, due to higher substrate specificity and efficiency, milder and easier controlled operating conditions, and fewer by‐products (Cheirsilp, Kaewthong, & H‐Kittikun, 2007; Feltes et al., 2010; Soumanou, Pérignon, & Villeneuve, 2012; Weber & Mukherjee, 2004). The enzymatic synthesis of MLM lipids includes one‐step and two‐step enzymatic hydrolyses. In one‐step hydrolysis, the products, which are collected in the form of unreacted triacylglycerols, are difficult to purify. Furthermore, this method is limited due to low rate of special fatty acid insertion (<50%) and high rate of acyl transfer (Akoh & Kim, 2008; He et al., 2017; He, Li, Kodali, Chen, & Guo, 2016; Kim & Akoh, 2015). On the other hand, two‐step enzymatic hydrolysis connects long‐chain fatty acids at the three acyl positions of glycerin skeleton in the synthesis of LLL triacylglycerols, and MLM lipids are obtained by special *sn*‐1,3 localized enzymatic hydrolysis (Del et al., 2009; Esteban et al., 2009; Solaesa, Sanz, Falkeborg, Beltran, & Guo, 2016). Compared with one‐step enzymatic hydrolysis, this method has higher rate of fatty acid insertion but lower rate of reaction (He, Li, Guo, & Chen, 2018). Therefore, the acceleration of enzymatic hydrolysis of acyl groups and the reduction of acyl group transfer by long‐term reaction are the key pathways for the synthesis of MLM lipids.

Ultrasonic treatment mainly uses power and cavitation to change or accelerate physical, chemical, and biological characteristics or states of substance (Fiametti et al., 2011; Li et al., 2005). Large amounts of foam produced during the ultrasonic treatment of reactants produce immense heat and pressure while causing dramatic collapse (Özbek & Ülgen, 2000). Consequential, microjets formed during treatment helps generate turbulence in the reaction system, thus accelerating the reaction frequency. Moreover, as an auxiliary treatment, the ultrasonic treatment can reduce the size of some oligomers, while increasing substrate surface area and action frequency of catalyst, thereby reducing limitations due to mass transfer (Bansode & Rathod, 2014; Guo et al., 2013; Lerin et al., 2014). Therefore, ultrasonic treatment is considered highly suitable for enzymatic synthesis of MLM lipids. Numerous reports have shown that ultrasonic treatment using a microtip probe produces more preferable results; consequently, it is employed to accelerate the rate of enzymatic esterification of phytosterol and flavonoids with fatty acids in organic solvent (Zheng et al., 2012, 2013). However, according to the literature, the effectiveness of this innovative ultrasonic treatment on enzymatic transesterification is limited. Therefore, a novel experimental approach is required to investigate the influence of the enzymatic method with ultrasonic treatment on the synthesis of MLM lipids.

To the best of our knowledge, a systematic study on enzymatic synthesis of MLM lipids with different types of fatty acid acyls, such as saturated, monounsaturated, and polyunsaturated fatty acids, and different ultrasound pretreatments has not been reported. Thus, the present work aims at evaluating the effect of ultrasonic treatment on the enzymatic transesterification of MLM lipids using five 2‐monoacylglycerols (2‐MAGs), which are distinct fatty acids at the *sn*‐2 position, containing palmitic acid, octadecanoic acid, oleic acid, eicosapentaenoic acid, and docosahexaenoic acid as *sn*‐2 acyl donors. A sequential experimental strategy was carried out to evaluate the effects of ultrasonic treatment conditions, substrate concentrations, reaction temperatures, reaction times, and molar ratios on the insert conversion of *sn*‐2 acyl position of MLM lipids. Furthermore, to study possible positive effects of this method, the influence of different ultrasonic treatments was also investigated.

## 2 EXPERIMENTAL PROCEDURES

### 2.1 Materials

Materials including 2‐monoolein and diolein standard, mixed fatty acid methyl ester (37 mixins), were purchased from Sigma‐Aldrich Chemical Co. The immobilized lipase from Rhizomucor miehei (Lipozyme RM‐IM) was purchased from Novozymes A/S. Dry ethanol, *n*‐hexane, acetone, chloroform, NaCl, absolute ethanol, and acetonitrile were of HPLC or analytical grade and were obtained from Fisher Scientific.

### 2.2 Two‐step enzymatic reactions

#### 2.2.1 Production of 2‐monoacylglycerols (2‐MAGs) by ethanolysis of triacylglycerols

**Figure 1:**
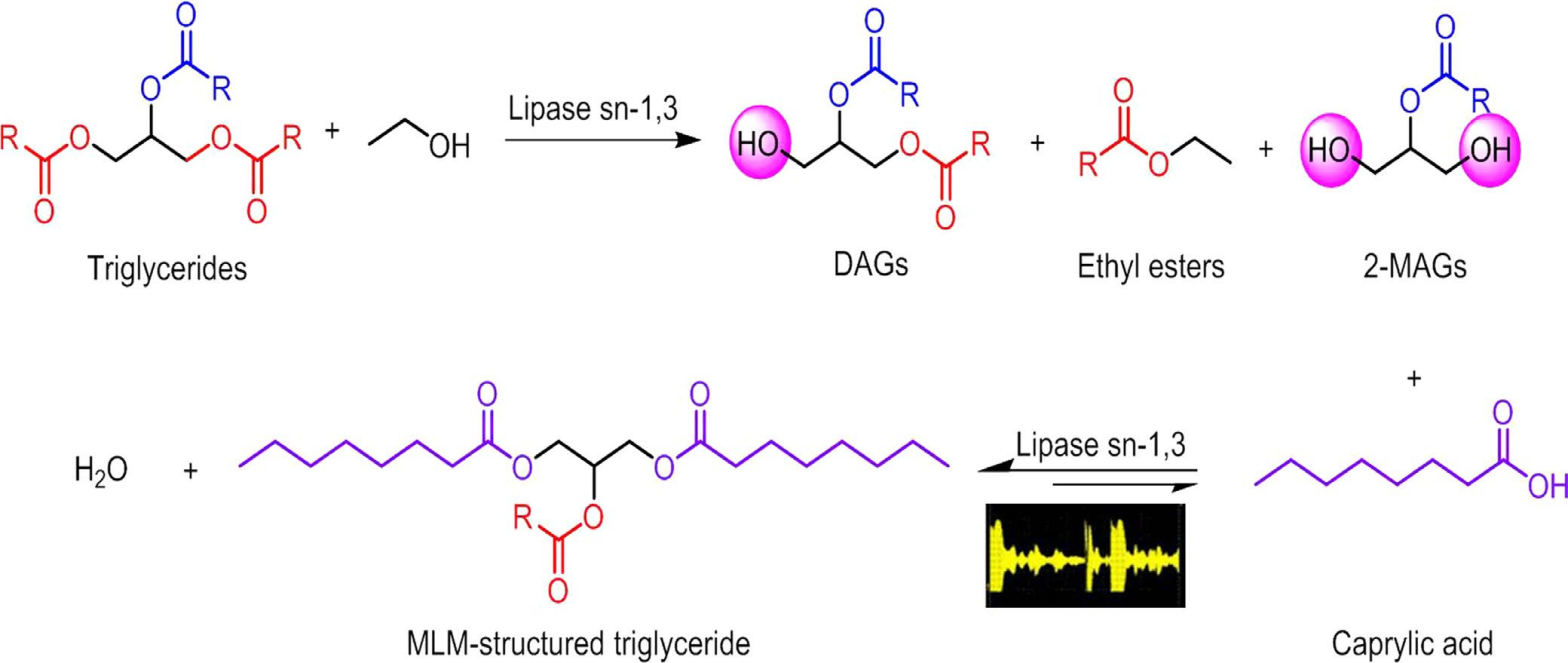
Two‐step enzymatic reaction for the synthesis of structured triacylglycerides

Medium–long–medium‐type structured triacylglycerols were produced by a two‐step enzymatic reaction according to the procedure proposed by Muñío, Robles, Esteban, González, and Molina (2009) with some modifications (Figure 1). A blend of algae oil, rapeseed oil, coconut oil, and palm oil (0.39:0.35:0.22:0.03, wt/wt/wt/wt) was equilibrated with 3 g of dry ethanol in a closed vessel with a water activity of 0.75 over saturated NaCl. The mixtures were then homogenized by magnetic stirring (IKA) at 200 rpm in a oil bath at 40°C for 15 min. Subsequently, 100 mg of immobilized Lipozyme RM‐IM was added, which triggered the reaction, during which a 10 μl sample was periodically withdrawn from the reaction mixtures for analysis by thin‐layer chromatography (TLC). After 5 hr, the mixtures were transferred to a centrifuge tube (Thermo Fisher Scientific Inc.), centrifuged at 7,100 × *g* for 6 min to remove the biocatalyst, and further analyzed by TLC.

The purification of 2‐MAGs was achieved according to the method proposed by Johnner, Tutus, Kusnandar, Hamidah, and Tursino, (2018) and Rodríguez et al. (2012) with some modifications. The mixture was extracted using ethanol:water (9:1 v/v) as solvent. It was then washed with *n*‐hexane, the upper layer containing esters and residuals of unreacted triglycerides was discarded, while the bottom layer was the hydroethanolic phase containing 2‐MAGs. The separated bottom layer was centrifuged at 1,700 × *g* for 6 min in a mixture of water:ethanol (9:1 v/v) and *n*‐hexane. The upper layer was the hexanic phase containing 2‐MAGs, which was separated, the residual ethanol/water was removed by a rotary evaporation apparatus (IKA), and 2‐MAG was recrystallized several times until TLC showed a single band.

#### 2.2.2 Production of MLM structured lipids by esterification of purified 2‐MAG with caprylic acid

Purified MAGs (100 mg) were added to caprylic acid (150 mg, 1:1.5 w/w), recycled Lipozyme TL‐IM (10 wt% by weight of MAGs and caprylic acid), *n*‐hexane (3 ml), and 4 Å molecular sieves (2 mg). The mixture was magnetically stirred (200 rpm; IKA) in the presence or absence of ultrasonic pretreatment (power: 50, 100, 150, 200, and 250 W; frequency: 20, 25, 30, 35, and 40 Hz). Meanwhile, different reaction parameters of mixture including reaction temperature (30, 35, 40, 45, 50, and 55°C), molar ratio of MAGs to CA, and reaction time (1, 2, 3, and 4 hr) were investigated. The lipase was removed via centrifuge at 2,700 ×*g*  for 10 min. The final lipid samples were isolated and stored at −20°C before purification, analysis, and characterization.

### 2.3 Purification of TAGs

Purification of TAGs was carried out following the procedure reported by Muñío et al. (2009). Lipase was removed by centrifugation, the organic phase was dried over anhydrous sodium sulfate, and solvent was removed by vacuum evaporation. The crude mixture was purified by column chromatography. A slurry of silica gel (10 g) and aluminum oxide (10 g) in 50 ml of *n*‐hexane was prepared and then poured into a column (300 × 30 mm). The reaction mixture (1 g) containing DAGs, fatty acids, TAGs, and 2‐MAGs was loaded onto the column and subsequently eluted with *n*‐hexane/diethyl ether (95/5, v/v) solution. The eluted fractions were recovered and analyzed by TLC and HPLC (Shimadzu).

### 2.4 Analysis of lipid class composition by TLC

The lipid classes (TAGs, DAGs, and MAGs) were analyzed by TLC following the procedure reported by Muñío et al. (2009). TLC plate was activated at 105°C for 1 hr before analysis. The samples (10 μl) were dissolved in 200 μl of *n‐*hexane/diethyl ether (84/16, v/v), and 1 μl of each diluted sample was placed on the activated TLC plate, which was then placed in an oven at 120°C to remove volatile solvents. Then, a mixture of *n‐*hexane:diethyl ether:formic acid (84/16/0.04, v/v/v) was spread onto the TLC plate containing the samples, and the fractions corresponding to different lipid classes were scraped from the TLC plates for further analysis.

### 2.5 Analysis of products by HPLC

HPLC analysis of products was conducted following the procedure described by Snehal, Jyotsna, Parag, and Satyanarayan (2018) with some modifications. TAGs were characterized by HPLC (Inertsil ODS‐2 column: 250 mm × 4.6 mm i.d.; particle size: 5 μm; Shimadzu) equipped with UV detector (set at 254 nm). The samples were dissolved in the mobile phase and injected onto the column at 2 µl injection volume. The mobile phase consisted of acetonitrile and acetic acid (9/1, v/v), and flow rate remained constant at 1 ml/min. The reaction was monitored using the molar or weight percentage of TAGs produced.

### 2.6 Analysis of *sn*‐2 fatty acid composition by GC‐FID

*sn*‐2 fatty acids were analyzed following the method proposed by Pande, Sabir, Baeshen, and Akoh (2013) with some modifications. After the reaction was complete, the mixtures were centrifuged at 7,100 *g* at 4°C for 5 min, and 1 ml of 6 M HCl solution was then added to terminate the reaction. The fatty acid composition of the samples was determined by GC (Shimadzu). Samples containing 2 ml of 0.5 M NaOH‐CH_3_OH were saponified at 60°C for 30 min and subsequently reacted with 14% boron trifluoride at 60°C for 5 min. After the reaction was complete, the fatty acid methyl ester was extracted with 2 ml of *n*‐hexane, and the molar percentage of fatty acid composition at *sn*‐2 position of the produced TAGs was calculated.

The fatty acid methyl ester was identified and quantified using GC‐17A Shimadzu gas chromatograph equipped with AOC‐5000 autosampler (Shimadzu) with 30 m × 0.32 mm Equity DB‐1 column. The flow rate of carrier gas was set at 1.0 ml/min, and split ratio was set at 100:0. Temperature of the injector and detector was fixed at 250°C. The initial temperature was set at 60°C for 3 min, increased to 175°C at a rate of 5°C/min, and held for 15 min; it was then increased to 220°C at a rate of 2°C/min and held for 10 min. According to the standard analysis of fatty acids, peak time and relative peak area were used for quantitative and qualitative analyses of fatty acid methyl ester.

### 2.7 Reusability of RM‐IM in MLM lipids

The reusability of RM‐IM was evaluated under optimal conditions. After each enzymatic hydrolysis (ethanol dissolution), the biocatalyst RM‐IM was removed from the samples by centrifugation at 7,100 *g* for 6 min. The supernatant, which was used to calculate yield of MLM, was washed three times with anhydrous ethanol/*n‐*hexane (1:1, v/v) and dried in a dryer equipped with a vacuum controller (VC 10, IKA) for 24 hr. During the esterification, the molecular sieves were removed using clean tweezers and activated for subsequent reactions.

### 2.8 Statistical analysis

Data calculation was carried out using Microsoft Excel 2010 and Microcal Origin 8.5. All tests and samples were conducted in triplicates, and the data were presented as means ± standard deviations (*n* = 3). The one‐way ANOVA and Duncan's range tests were applied to determine significant differences between means (*p* < 0.05).

## 3 RESULTS AND DISCUSSION

### 3.1 Effects of ultrasonic treatment on the conversion of MLMs

**Figure 2:**
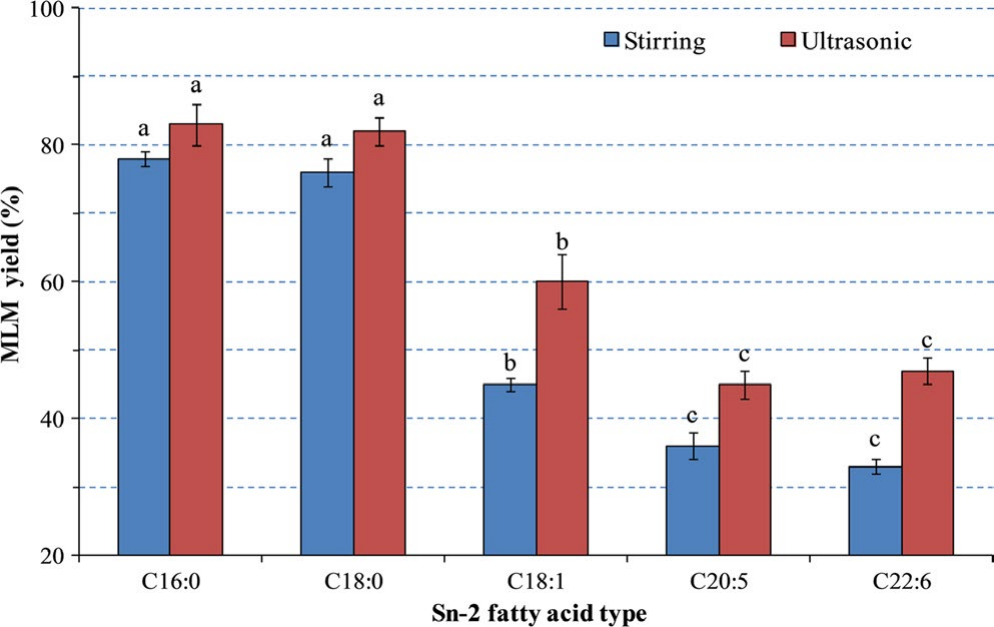
Effects of ultrasonic and conventional treatment on the synthesis of MLM lipids with different fatty acids as *sn*‐2 acyl donors. Different superscript letters indicate the significant difference between conventional and ultrasonic treatment (*p* < 0.05)

The enzymatic synthesis of MLM lipids is considered an effective and green method (Tang, Wang, Huang, Jin, & Wang, 2015). In this study, the effect of ultrasonic treatment on the synthesis rate of MLM lipids during enzymatic hydrolysis was studied (Figure 2). Considering different fatty acid acyl donors, the synthesis rates of C16:0 and C18:0 lipids (accounted for 78.20%), which were inserted at the *sn*‐2 position of MLM, were higher than (*p* < 0.05) those of unsaturated fatty acids, such as C18:1 (46.8%). Additionally, the synthesis rate of MLM decreased with increasing degree of unsaturation of fatty acid acyl donors. Similar results, which showed relatively low content of unsaturation of fatty acid in the *sn*‐2 position of the produced TAG, were also reported (Morales‐Medina, Munio, Guadix, & Guadix, 2017). This was as a result of unsaturated fatty acids having lower probability of contact with catalytic enzymes, due to steric hindrance of their double bonds, thereby hindering addition at the *sn*‐2 acyl position of MLMs (Bimbo & Breivik, 2007). Compared with conventional enzymatic hydrolysis, ultrasonic treatment accelerated the enzyme‐catalyzed reaction, hence improving the synthesis rate of MLM lipids. This treatment aids attachments of C16:0 and C18:0, as well as unsaturated fatty acids, such as C18:1 (60.1%), C20:5 (43.1%), and C22:6 (45.4%), to the *sn*‐2 position of TAG. Based on these results, the effects of ultrasonic intensity, ultrasonic frequency, oil and ethanol molar ratio, reaction time, and reaction temperature on the synthesis of MLM lipids were further examined. Additionally, the mechanism in which different fatty acids were selectively inserted into the *sn*‐2 acyls of MLM lipids under ultrasonic treatment was determined.

### 3.2 Effects of ultrasonic intensity

**Figure 3:**
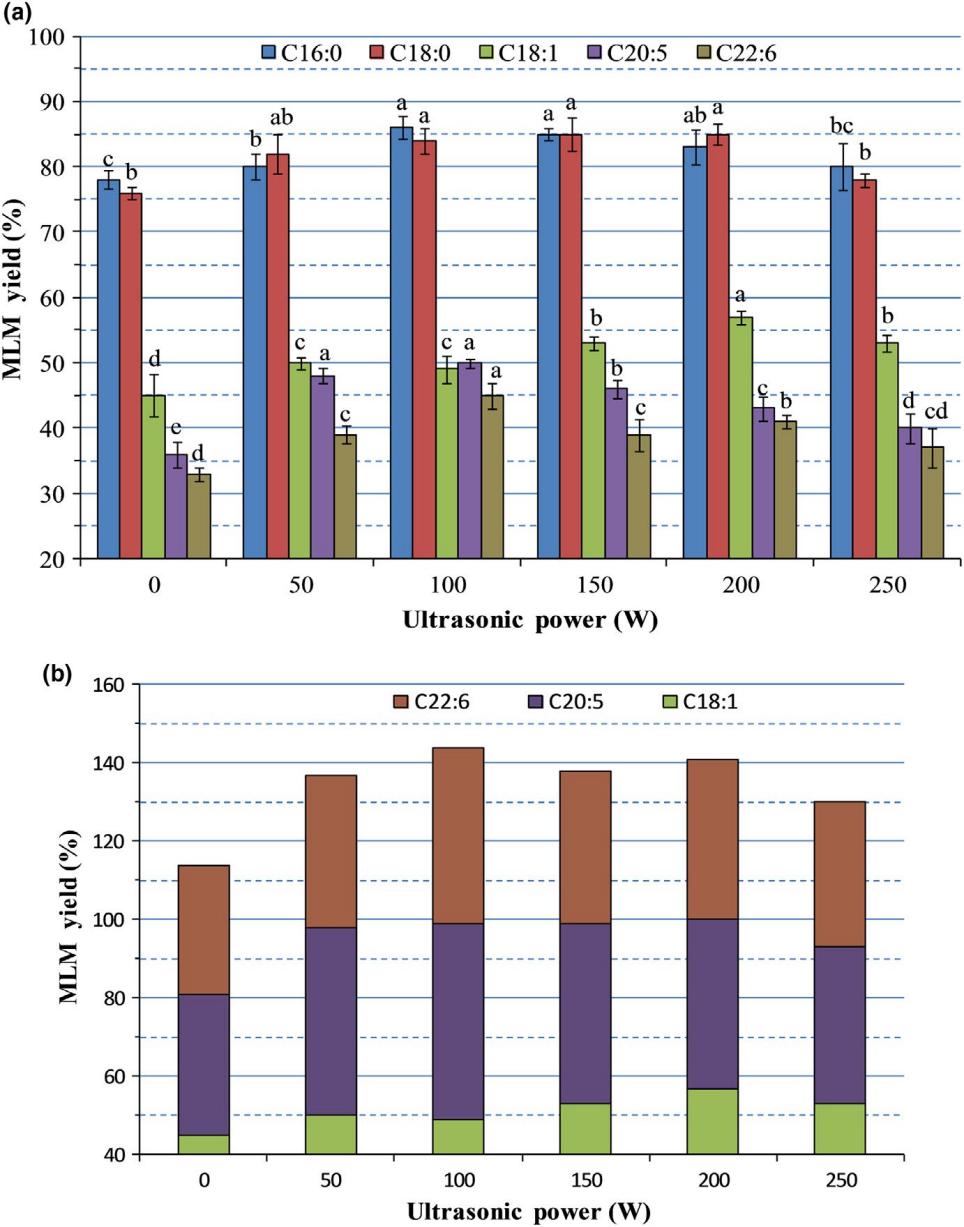
(A) Effects of ultrasonic intensity on the synthesis of MLM lipids with different fatty acids as *sn*‐2 acyl donors. Different superscript letters indicate the significant difference between different MLM yields treated with different ultrasonic intensities (*p* < 0.05). (B) Effects of ultrasonic intensity on the synthesis of MLM lipids with unsaturated fatty acids as *sn*‐2 acyl donors

Figure 3A shows the insertion rates of different fatty acids at the *sn*‐2 acyl position of MLM lipids under various ultrasonic intensities of 0–250 W. The insertion rates of C16:0 (78.3%) and C18:0 (76.4%) were substantially higher than (*p* < 0.05) those of unsaturated fatty acids (C18:1 [39.6%], C20:5 [36.1%], and C22:6 [33.7%]) under nonultrasonic conditions (0 W). The insertion rates of various fatty acids at the acyl group increased with increasing ultrasonic intensity from 50 to 150 W. The maximum insertion rate of C16:0, which was about 10.4% higher than the minimum insertion rate obtained at 0 W, was achieved at 100 W. Moreover, the insertion rate of C18:1 obtained under 200 W ultrasonic intensity was 13.2% higher than that of conventional treatment (*p* < 0.05). In addition, the insertion rate of unsaturated fatty acids was lower than that of saturated fatty acids. The results showed that the ultrasonic intensity was not selective to specific types of fatty acids. Compared with the effects caused by low ultrasonic intensity, high ultrasonic intensity had considerable effect on MLM lipid synthesis. The conversion rate of MLM showed a partial increase with increasing ultrasonic power from 0 to 150 W. However, an extremely high ultrasonic intensity promoted enzyme degradation, thereby causing a substantial decrease in conversion rate, as observed when the power was higher than 200 W. Additionally, high ultrasonic intensity may breakdown the enzyme, causing denaturing, that can be accompanied by a decrease in enzymatic activity, which is the major reason why the conversion rate decreased under high ultrasonic intensity (Özbek & Ülgen, 2000; Trentin et al., 2015).

As shown in Figure 3B, ultrasonic treatment influences the insertion of different fatty acids at the *sn*‐2 acyl position of MLM lipids, as demonstrated by the enhanced synthesis rates of unsaturated fatty acids in the structured lipids with different ultrasonic intensities. The ultrasonic treatment at 100 W intensity increased (*p* < 0.05) the synthesis rate of saturated fatty acid by 28.2% compared with the conventional enzymatic reaction. Although the synthesis rates decreased at higher ultrasonic intensity, they remained higher than those obtained from the conventional enzymatic reaction. These results showed that ultrasonic‐assisted reactions enhanced the synthesis rates of structured lipids with unsaturated fatty acids, which may be due to the ultrasonic waves enhancing enzymatic activity (Brenelli & Fernandes, 2003). Zheng et al. (2013) also observed that enzymatic esterification reaction rapidly increased with increasing power within the range of 50–200 W. Moreover, as they further increased the ultrasonic intensity, enzymatic acidity was inhibited; however, this only affected the maximum enzymatic rate. Other reports have described similar results, in which increased ultrasonic power within a certain range can enhance enzymatic reaction rate (Pingret, Fabiano‐Tixier, & Chemat, 2013). Therefore, we concluded that high‐intensity ultrasonic treatment was beneficial to the insertion of unsaturated fatty acids into MLM lipids.

### 3.3 Effects of ultrasonic frequency

**Figure 4:**
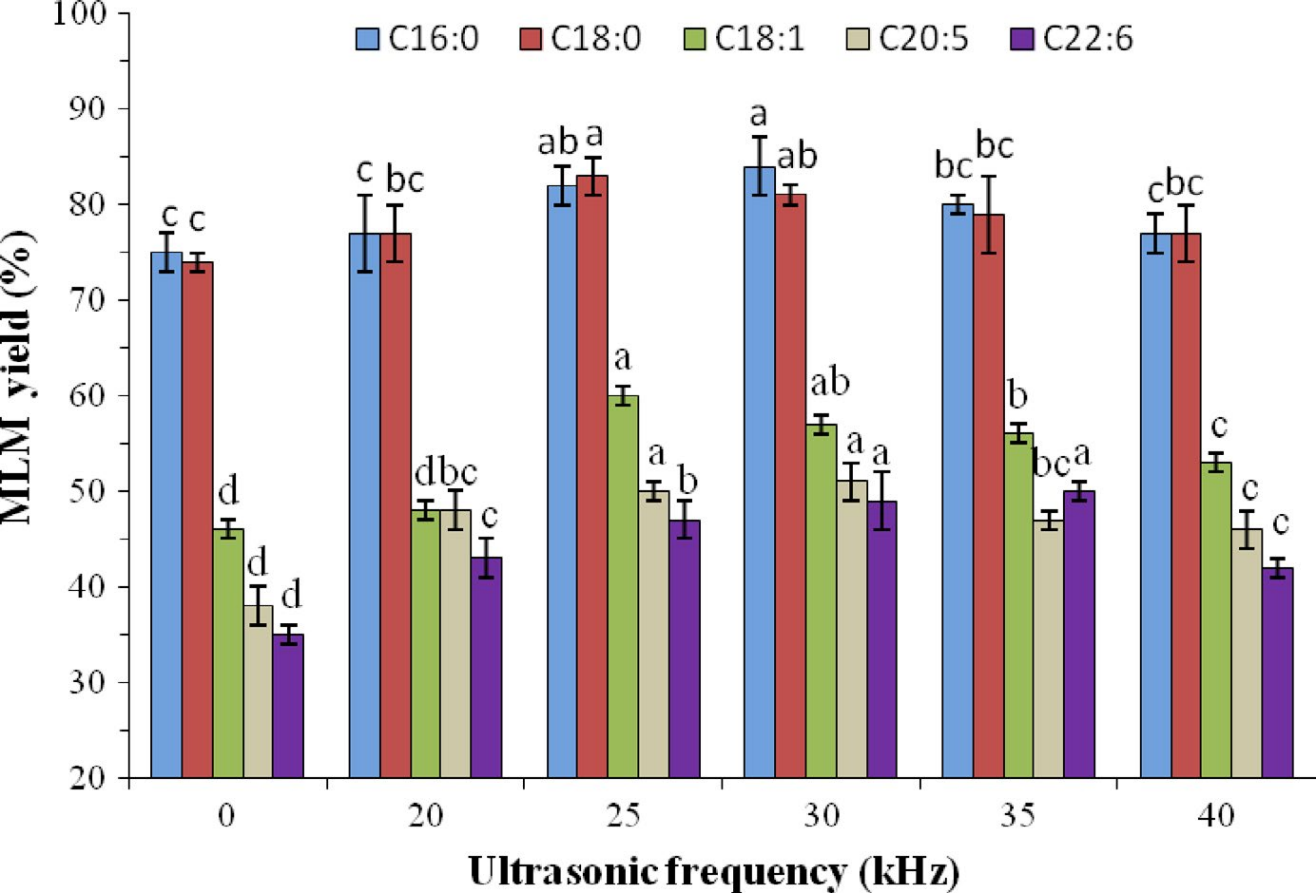
Effects of ultrasonic frequency on the synthesis of MLM lipids with different fatty acids as *sn*‐2 acyl donors. Different superscript letters indicate the significant difference between different MLM yields treated with different ultrasonic frequencies (*p* < 0.05)

Ultrasonic frequency and intensity are equally important factors influencing the synthetic ratio of MLM structured lipids. The influence of ultrasonic frequency at the range of 20–40 kHz at ultrasonic power of 150 W on the synthetic ratio of MLM structured lipids was investigated. As presented in Figure 4, the synthesis rates of structured lipids from various fatty acids increased with increasing ultrasonic frequency, reaching a maximum value at frequencies 25 and 30 kHz. In contrast, by further increasing ultrasonic frequency, an increase in the synthesis rate of the structured lipids (*p* > 0.05) was not observed. Li et al. (2005) showed that the reaction rate increased with increasing ultrasonic frequency, but the degree of increase can change only within a certain time period. Therefore, it was possible that enzymatic activity was stimulated below 20 kHz ultrasonic treatment, while partially deactivated by other physical effects, such as shear forces and cavitation (Lerin et al., 2014). Therefore, 25 kHz frequency was chosen in subsequent experiments studying the features of lipase‐catalyzed reactions.

### 3.4 Effects of ultrasonic treatment time

**Figure 5:**
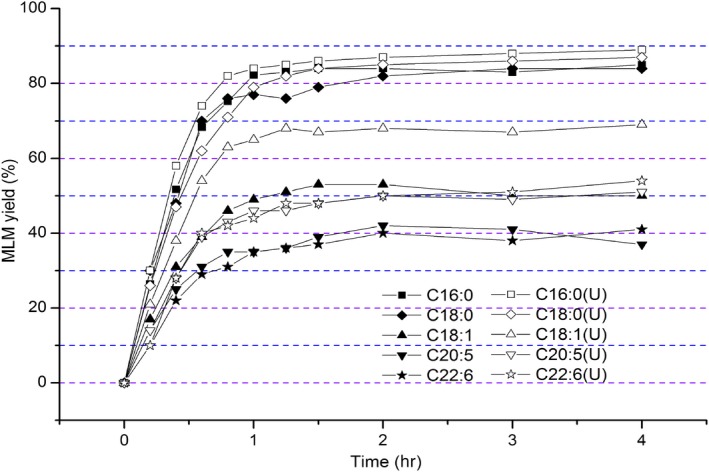
Effects of ultrasonic time on the synthesis of MLM lipids with different fatty acids as *sn*‐2 acyl donors

Ultrasonic technology is a new technology widely used in various sample preparations. The induction effect of ultrasonic treatment on biological materials is generally divided into thermal and nonthermal effects. When an ultrasonic energy is transmitted to an attenuating medium, part of the energy is converted into heat, which can act as an additional heat source. Compared with the conventional enzymatic method, ultrasonic‐assisted methods effectively improved the synthesis rates of structured lipids (Figure 5). Within the first hour of the enzymatic reaction, the synthesis rates of all structured lipids with different fatty acids rapidly increased. The conversion rates of C16:0 and C18:0 undergone different ultrasonic treatments reached 74.3% and 71.7%, respectively (*p* < 0.05), which were higher than those of other fatty acid acyl groups. Additionally, the conversion rate of C18:1 (57.3%) undergone ultrasonic treatment was considerably higher than (*p* < 0.05) that of other unsaturated fatty acids (C20:5 [45.8%] and C22:6 [43.2%]). These results indicate that the effect of ultrasonic treatment on the fatty acids at the *sn*‐2 acyl position of structured lipids can be ranked as follows: saturated fatty acids > unsaturated fatty acids (low degree of unsaturation) > unsaturated fatty acids (high degree of unsaturation). The synthesis rates obtained under the conventional method remained stable, although with extended reaction time. In contrast, the ultrasonic‐assisted method improved the synthesis rates by 20%–27%. Similar results, which revealed that the enzyme exhibits maximum activity after ultrasound pretreatment, were also reported (Fernandez‐Lafuente, 2010; Liu et al., 2015). These observations indicate that ultrasonic treatment has positive effect on the initial synthesis rate of structured lipid at its initial stage; however, such effect is not long‐term. The results are consistent with those previously reported, in which ultrasound effectively reduced particle size of the substrate while increasing the surface area of the enzyme—the two factors that can overcome the limitations due to mass transfer (Bansode & Rathod, 2017). Despite such behavior, it is especially important when enzymes are used to catalyze reactions in organic solvents. Therefore, extended high‐intensity ultrasonic time produces immense heat, thereby reducing enzymatic activity.

### 3.5 Effects of enzyme and substrate molar ratio

**Figure 6:**
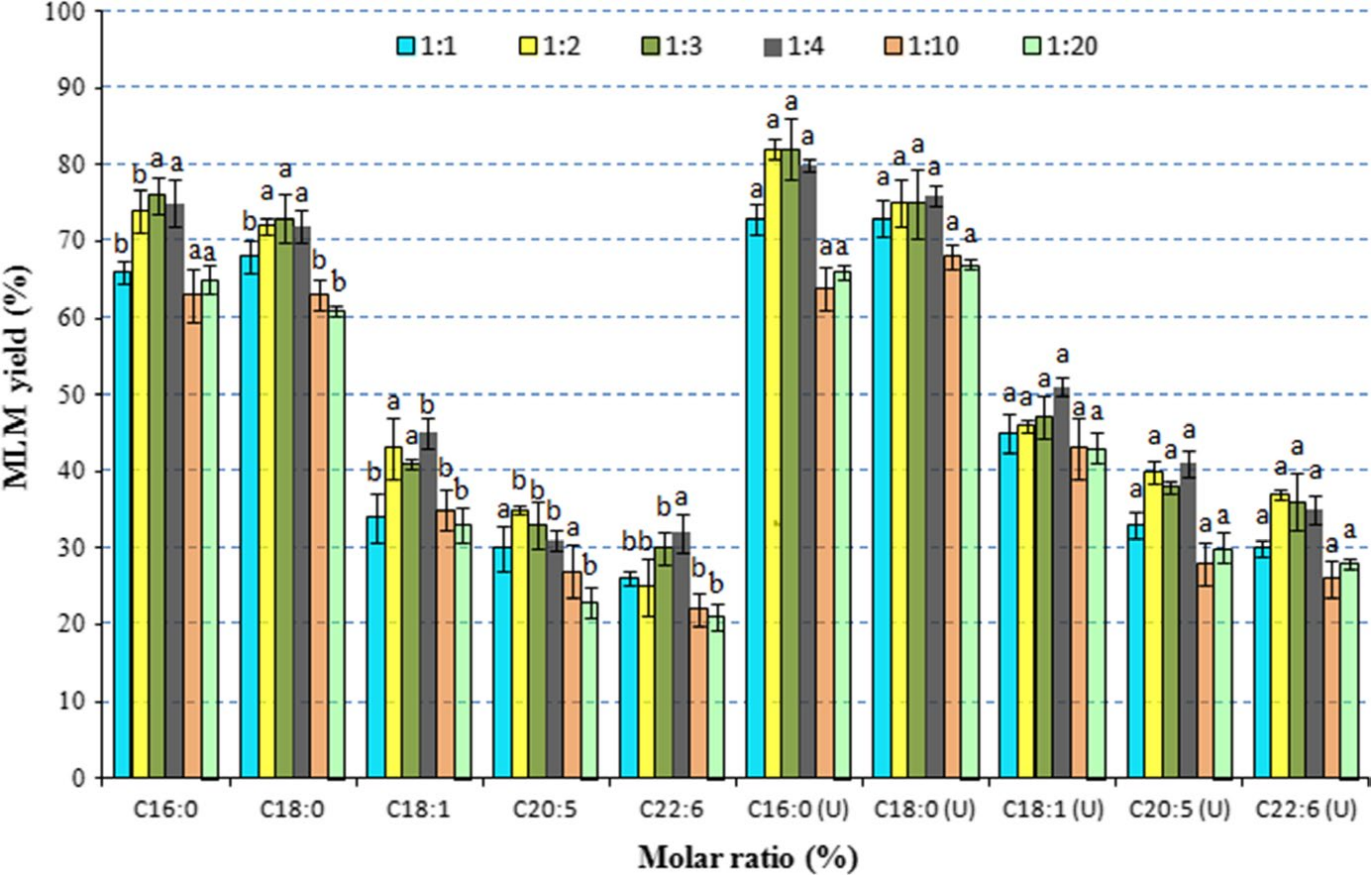
Effects of molar ratio on the synthesis of MLM structural lipid with different fatty acids as *sn*‐2 acyl donors. Different superscript letters indicate the significant difference between different MLM yields treated with different molar ratios (*p* < 0.05)

The molar ratio of enzyme and substrate plays an important role in enzymatic reactions. An appropriate ratio of lipase can increase the reaction rate, thereby decreasing the reaction time (Huang et al., 2012). However, by increasing the amount of costly lipase, it leads to an increase in total production cost. This study compares the synthesis rates of MLM lipids at various molar ratios of enzyme and substrate (Figure 6). The synthesis rates of both saturated and unsaturated fatty acids at the *sn*‐2 position of structured lipid increased with increasing enzyme:substrate molar ratio from 1:1 to 1:3 (*p* < 0.05). The synthesis rate of fatty acid acyl at 1:4 molar ratio was comparable to 1:3 molar ratio (e.g., C20:5). However, the synthesis rate decreased when the molar ratio exceeded 1:4, specifically at 1:10 or 1:20 ratios. Therefore, reduction lipase concentration may cause aggregation on the substrate's surface, thus reducing contact area between enzyme (lipase) and substrate (Liu et al., 2016).

Under both conventional and ultrasonic‐assisted enzymatic methods, the synthesis rates of MLMs from acyl donors C16:0 and C18:0 were not significantly changed. Nevertheless, the synthesis rate of MLM using unsaturated fatty acids as *sn*‐2 acyl donors under ultrasonic‐assisted enzymatic method was significantly higher than that of conventional method. Hence, the synthesis of MLM lipids using unsaturated fatty acids as *sn*‐2 acyl donors can be synergistically stimulated by ultrasonic treatment and substrate molar ratio. We hypothesized that the ultrasonic wave accelerates the contact frequency between lipase and unsaturated fatty acid, thereby reducing the overload of substrate lipase surface. Additionally, it may increase the contact frequency between unsaturated fatty acid and glycerol skeleton, promoting an increase in synthesis rate of MLM lipids (Bansode & Rathod, 2017). In addition, relative activity of enzymes highly depends on the interaction between substrate and its binding sites on enzyme. Thus, the environment of the catalytic pocket of lipase may be altered upon increasing fatty acid concentration.

### 3.6 Effects of temperature

**Figure 7:**
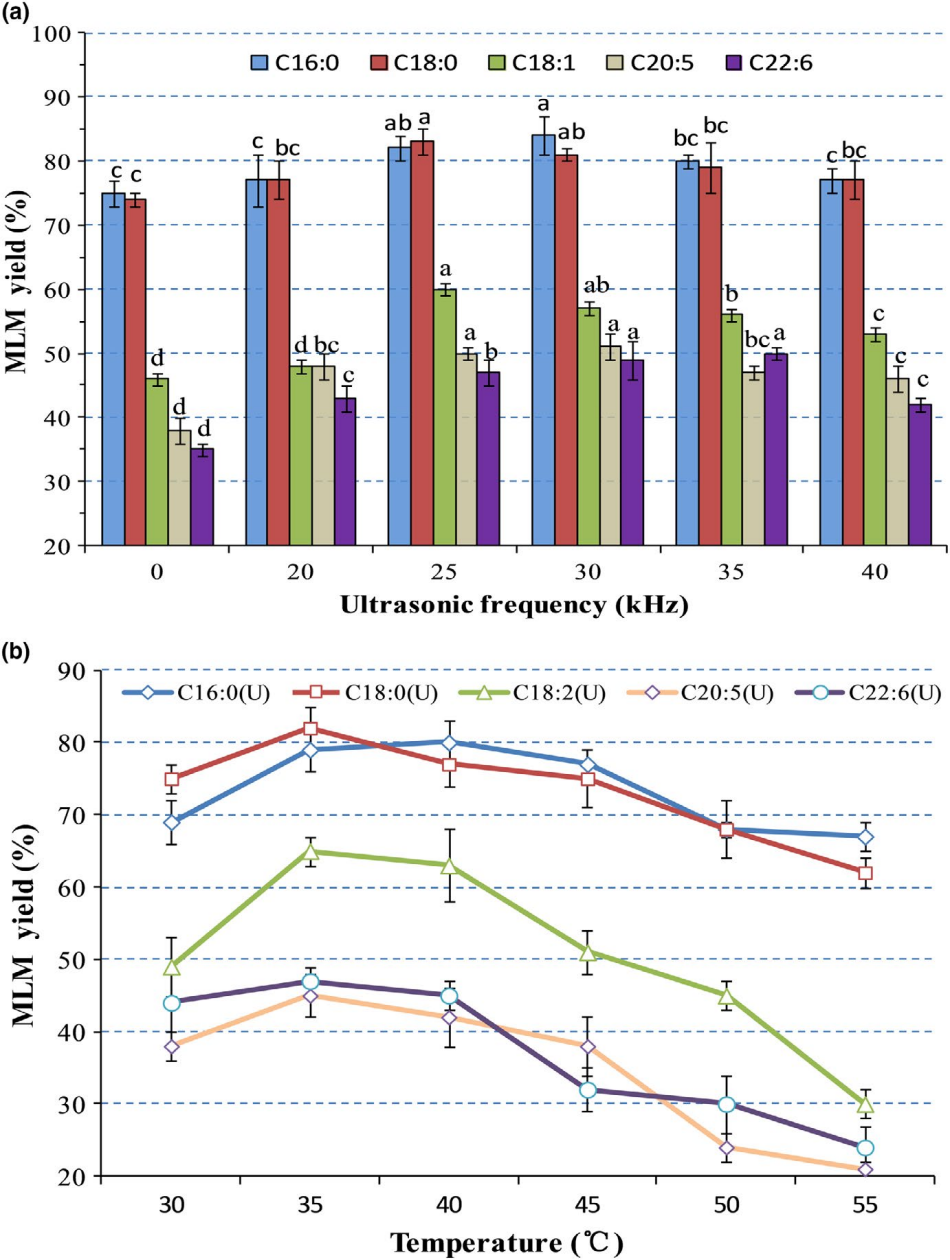
(A) Effects of temperature on the synthesis of MLM lipids with different fatty acids as *sn*‐2 acyl donors under conventional treatment. (B) Effects of temperature on the synthesis of MLM lipids with different fatty acids as *sn*‐2 acyl donors under ultrasonic pretreatment

The effects of reaction temperature, ranging from 30 to 55°C, on the lipase‐catalyzed esterification of MLM lipids were investigated (Figure 7A). In the case of conventional enzymatic hydrolysis, the conversion rate of structured lipids gradually increased with increasing reaction temperature from 30 to 40°C. Upon reaching 55°C, the conversion rates substantially decreased (*p* < 0.05), especially those of C16:0 and C18:0. Additionally, the synthesis rates of MLM lipid from unsaturated fatty acids were considerably lower than those of MLM lipid from saturated fatty acids (*p* < 0.05). As shown in Figure 7B, with ultrasonic treatment, the optimum reaction temperature for all types of fatty acids was 35°C. This indicates that ultrasonic treatment reduces the optimum reaction temperature. The synthesis rates of lipids from unsaturated fatty acids obtained under ultrasonic treatment were remarkably higher than those of conventional enzymatic hydrolysis. Zhang et al. (2017) showed that as the temperature increased from 25 to 40°C, an increase in MAGs content from 27.19% to 28.39%, respectively, occurs without significant differences. Therefore, ultrasonic treatment may increase enzyme solubility or partially increase the reaction temperature. By increasing the temperature, the synthesis rates of unsaturated fatty acids at the *sn*‐2 acyl sites of MLM lipids decreased, which may be due to heat caused by ultrasonic treatment. These results showed that the optimum and inactivation temperatures of ultrasonic‐assisted lipase were approximately 5°C lower than those of conventional mechanical agitation. As stated earlier, when ultrasonic energy is transmitted to an attenuating medium, some of the energy can be converted into heat, which can act as a heat source (Deenu, Naruenartwongsakul, & Kim, 2013; Parniakov et al., 2015). Hence, ultrasonic treatment increases the reaction temperature, while reducing the optimal reaction temperature and decreasing reaction time.

### 3.7 Reusability of Lipozyme

**Figure 8:**
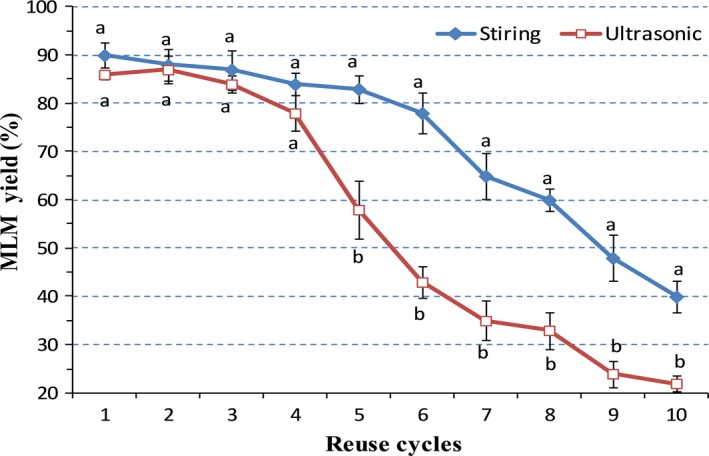
Reusability of the Lipozyme RM‐IM used in the synthesis of MLM lipids under ultrasonic and conventional treatment. Different superscript letters indicate the significant difference between different reusability of the Lipozyme RM‐IM treated with conventional and ultrasonic treatments (*p* < 0.05)

Reusability and recoverability of lipase are crucial factors determining its applicability in biological technology (Nielsen, Rancke‐Madsen, Holm, & Burton, 2016). To evaluate the effect of ultrasonic treatment on the applicability of lipase in industries, the stability of Lipozyme RM‐IM was determined by measuring its structured lipid content obtained during continuous enzymatic hydrolysis under optimum conditions. Figure 8 shows that the conversion rate of structured lipid remained at 75.3% after 6 cycles in the absence of ultrasonic treatment. In contrast, with ultrasonic treatment, the conversion rate of structured lipid considerably declined (*p* < 0.05) from 31.10% to 26.90% after the fourth cycle, and the amount of enzyme after usage decreased compared with those without ultrasonic treatment. This indicates that ultrasound may disrupt the structure of lipase, in turn reducing activity and shortening its service life in the reaction. These observations are, however, inconsistent with those reported by Akanbi, Sinclair, and Barrow (2014) and Lerin et al. (2014), which may be attributed to different types and selectivities of enzymes used in the reaction.

### 3.8 Insertion of different fatty acids into the *sn*‐2 acyl sites of mixed lipids

Under specified conditions (150 W ultrasonic intensity, 10% enzyme loading, 60°C temperature, and 4‐hr reaction time), the effects of mechanical stirring (nonultrasonic treatment) and ultrasonic treatment on the insertion rates of different fatty acids into the *sn*‐2 acyl site of mixed lipids were examined. The experiment was conducted to explore the mechanism of how different fatty acids were inserted into the *sn*‐2 acyl of MLM lipids. Mixtures of lipid sample containing algae oil, rapeseed oil, coconut oil, and palm oil (0.39:0.35:0.22:0.03, wt/wt/wt/wt) ratio were prepared, and the proportions of saturated fatty acids (SFAs), monounsaturated fatty acids (MUFAs), and polyunsaturated fatty acids (PUFAs) were compared. Table 1 shows the fatty acid composition of MLM lipid obtained from the mixed oil system under conventional and ultrasonic treatments. Under conventional treatment, SFAs were more easily inserted into the *sn*‐2 acyl sites of structured lipids, compared with MUFAs and PUFAs. We speculated that unsaturated fatty acids’ double bonds affect the steric hindrance of the enzyme, thus reducing the synthesis rate (Tan & Yin, 2005).

**Table 1:** Major fatty acid composition resulting from conventional and ultrasonic treatments of MLM lipid synthesis

Fatty acid	Mixed oil^A^ (mol%)	Conventional (mol%)	Ultrasonic (mol%)			
			I^B^	II^C^	III^D^	
C14:0	6.2 ± 0.31^a^	5.2 ± 0.48^b^	5.8 ± 0.26^ab^	3.2 ± 0.28^d^	4.4 ± 0.42^c^	
C16:0	20.6 ± 1.35^bc^	21.1 ± 1.75^bc^	28.9 ± 2.58^a^	29.4 ± 1.44^a^	23.2 ± 2.03^b^	**↓**
C16:1	14.6 ± 0.87^ab^	13.2 ± 0.99^bc^	11.4 ± 1.24^c^	15.4 ± 1.13^a^	14.7 ± 1.34^ab^	
C18:0	4.2 ± 0.34^d^	3.9 ± 0.36^d^	8.6 ± 0.18^a^	6.3 ± 0.26^c^	7.5 ± 0.16^b^	**↓**
C18:1	14.1 ± 0.78^a^	13.3 ± 0.49^a^	12.3 ± 0.22^b^	10.4 ± 0.53^c^	11.9 ± 0.74^b^	
C18:2	9.3 ± 0.30^a^	8.9 ± 0.35^a^	5.6 ± 0.32^b^	3.3 ± 0.19^d^	4.5 ± 0.33^c^	↘↗
C18:3	1.0 ± 0.14^a^	0.6 ± 0.52^ab^	NF	0.7 ± 0.16^ab^	0.5 ± 0.14^c^	
C20:4	3.9 ± 0.12^ab^	3.5 ± 0.36^bc^	2.4 ± 0.14^d^	4.2 ± 0.26^a^	3.2 ± 0.24^c^	
C20:5	8.2 ± 0.97^a^	7.7 ± 0.28^a^	6.1 ± 0.57^c^	6.4 ± 0.44^bc^	7.4 ± 0.22^ab^	**↑**
C22:5	1.3 ± 0.23^a^	1.0 ± 0.16^ab^	0.4 ± 0.13^c^	0.8 ± 0.12^b^	NF	
C22:6	18.5 ± 0.55^a^	16.9 ± 0.93^ab^	12.3 ± 1.15^c^	15.6 ± 1.13^b^	16.8 ± 0.86^ab^	**↑**
Other	4.8 ± 0.44^b^	2.9 ± 0.54^c^	6.2 ± 0.25^a^	4.3 ± 0.25^b^	5.9 ± 0.34^a^	
∑SFA	30.9 ± 2.53^c^	30.0 ± 3.10^c^	43.3 ± 3.34^a^	38.9 ± 2.66^ab^	35.1 ± 2.53^bc^	**↓**
∑MUFA	28.7 ± 2.95^a^	26.9 ± 1.85^ab^	23.7 ± 1.79^b^	25.8 ± 2.37^ab^	26.6 ± 2.65^ab^	**↑**
∑PUFA	40.3 ± 2.75^a^	40.3 ± 4.26^a^	26.8 ± 2.53^b^	31.0 ± 3.42^b^	32.4 ± 3.04^b^	**↑**

Note

Different superscript lowercase letters in the same row indicate significant differences (*p* ˂ 0.05). ^A^Mixed oil is a mixture of algae oil, rapeseed oil, coconut oil, and palm oil (0.39:0.35:0.22:0.03, wt/wt/wt/wt). Optimized reaction conditions are as follow: enzyme loading (10%); temperature (60°C); and reaction time (4 hr). I^B^ refers to ultrasonic frequency of 20 KHz with a power of 150 W. II^C^ refers to ultrasonic frequency of 25 KHz with a power of 150 W. III^D^ refers to ultrasonic frequency 30 KHz with a power of 150 W. “NF” represents “not found”; “↓” represents “decrease in MLM lipid synthesis with increasing ultrasonic frequency from 20 to 30 KHz”; “↑” represents “increase in MLM lipid synthesis with increasing ultrasonic frequency from 20 to 30 KHz.”

Compared with conventional treatment method, low‐frequency ultrasonic treatment remarkably improved the insertion rate of PUFA (4.5%) into the *sn*‐2 position of structured lipids. An increase in ultrasonic frequency promoted an increase in the synthesis rates of MUFA and PUFA from 25.8% and 31% to 27.1% and 33.2%, respectively. On the other hand, the synthesis rate of SFAs under high‐frequency ultrasonic treatment decreased (*p* < 0.05) considerably from 38.9% to 33.4%, which was 9.6% lower than conventional treatment. These results indicate that ultrasonic treatment accelerated the esterification and synthesis of different lipid acyl donors; thus, relatively high synthesis rates of structured lipid were obtained. In addition, the ultrasonic treatment improved the insertion rates of MUFAs and PUFAs into the *sn*‐2 position of structured lipids. Therefore, ultrasonic treatment accelerates the contact frequency between lipase and reactive surface of unsaturated fatty acids and carbon skeleton chain, thereby reducing contact with lipase. Similarly, contact frequency between different acyl donors (SFAs and unsaturated fatty acids) and glycerin skeleton chain increased, causing insertion rate of unsaturated fatty acids into the *sn*‐2 position to increase (Tan & Yin, 2005).

Furthermore, we observed that the insertion rates of MUFAs and PUFAs were lower than those of SFAs regardless of ultrasonic treatment (Table 1). It appeared that unsaturated fatty acids’ double bonds caused steric hindrance; as a result, the insertion at the *sn*‐2 of MLM lipids required additional energy and power. Zhang et al. (2018) observed that ultrasonic pretreatment improved the reaction rate while greatly enhancing affinity between substrate and lipase. Therefore, ultrasonic treatment, which can be easily controlled, can also reduce steric hindrance caused by unsaturated fatty acids, thus improving the synthesis rates of structured lipids. In addition, within the scope of ultrasonic frequency and power, the content of C16:0, C18:0, C20:5, and C22:6 increased, whereas that of C18:1 decreased initially but increased thereafter. These results suggest that ultrasonic treatment improves the insertion rates of long‐chain fatty acids; however, more comprehensive experiments should be performed to determine its mechanism in greater details.

## 4 CONCLUSION

In summary, a study was conducted to evaluate the effect of ultrasonic treatment conditions, which included substrate concentration, reaction temperature, reaction time, and molar ratio, on the insertion rate of fatty acids into the *sn*‐2 acyl position of MLM lipids. To evaluate the effects of ultrasonic treatment on the lipase‐catalyzed synthesis of MLM lipids from different fatty acids, we concluded the following: With increasing ultrasonic intensity, the activity and solubility of lipase increased, the reaction temperature was partially increased resulting in reduced reaction time, and the synthesis rate of MLM lipids, in which fatty acids were inserted into their *sn*‐2 acyl position, improved.

In the conventional enzymatic reaction, unsaturated fatty acids reduced the probability of contact between enzyme and substrate, due to the steric hindrance of double bonds, thereby limiting their attachment to the *sn*‐2 acyl of MLM lipids. In contrast, ultrasonic treatment increased the contact frequency between lipase and unsaturated fatty acids, thus reducing the loading on lipase and eliminating the effects of steric hindrance caused by unsaturated fatty acids. Additionally, ultrasonic treatment enlarged the contact areas between different *sn*‐2 acyl donors (SFAs and unsaturated fatty acids) and glycerin carbon skeleton, thereby increasing mass transfer during the synthesis of MLM lipids. This work opens up new opportunities for the use of ultrasonic technology in the synthesis of MLM lipids using different fatty acids as *sn*‐2 acyl donors.

## CONFLICT OF INTEREST

There are no conflicts to declare.

## ETHICAL REVIEW

This study does not involve any human or animal testing.

## INFORMED CONSENT

This study was not performed on any participants.
